# Hall conductance and topological invariant for open systems

**DOI:** 10.1038/srep06455

**Published:** 2014-09-24

**Authors:** H. Z. Shen, W. Wang, X. X. Yi

**Affiliations:** 1 Center for Quantum Sciences and School of Physics, Northeast Normal University, Changchun 130024, China; 2 School of Physics and Optoelectronic Technology, Dalian University of Technology, Dalian 116024, China

## Abstract

The Hall conductivity given by the Kubo formula is a linear response of quantum transverse transport to a weak electric field. It has been intensively studied for quantum systems without decoherence, but it is barely explored for systems subject to decoherence. In this paper, we develop a formulism to deal with this issue for topological insulators. The Hall conductance of a topological insulator coupled to an environment is derived, the derivation is based on a linear response theory developed for open systems in this paper. As an application, the Hall conductance of a two-band topological insulator and a two-dimensional lattice is presented and discussed.

Topological insulators (TIs) were theoretically predicted to exist and have been experimentally discovered in^1^,^2^,^3^, they are materials that have a bulk electronic band gap like an ordinary insulator but have protected conducting topological states(edge states) on their surface. In the last decades, these topological materials have gained many interests of scientific community for their unique properties such as quantized conductivities, dissipationless transport and edge states physics^4^,^5^. Although the exploration of topological phases of matter has become a major topics at the frontiers of the condensed matter physics, the behavior of TIs subject to dissipative dynamics has been barely explored. This leads to a lack of capability to discuss issues such as their robustness against decoherence, which is crucial in applications of the materials in quantum information processing and spintronics.

Most recently, the study of topological states was extended to non-unitary systems^6^,^7^,^8^, going a step further beyond the Hamiltonian ground-state scenario. This first step was taken with specifically designed dissipative dynamics described by a quantum master equation. Such an approach was originally proposed as a means of quantum state preparation and quantum computation^9^, which relies on the engineering of the system-reservoir coupling. To define the topological invariant for open systems, the authors use a scheme called purification to calculate quantities of quantum system in mixed states. To be specific, for a density matrix *ρ* in a Hilbert space 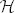, the density matrix *ρ* can be purified to |Φ*^ρ^*〉 by introducing an ancilla acting on a Hilbert space 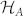 such that the tracing over the ancilla (Tr_A_) yields the density matrix, *ρ* = Tr*_A_*|Φ*^ρ^*〉〈Φ*^ρ^*|. In other words, mixed states can always be seen as pure states of a larger system (i.e., the system plus the introduced ancilla), the topological invariant (called Chern value in Ref. 7, 8) can then be defined as usual(closed system) TIs.

Turn to the topological invariant for closed system in more details. The topological invariant was first derived by Thouless *et al.*^10^,^11^, which provides a characterization of fermionic time-reversal-broken (TRB) topological order in two spatial dimensions. This was done by linear response theory in such a way that the Hall conductivity is represented in terms of a topological invariant (or the Chern number), which is related to an adiabatic change of the Hamiltonian in momentum space. However, the extension of this topological invariant from closed to open systems^7^,^8^ is not given in this manner to date, i.e., it is defined neither via the Hall conductance, nor by the linear response theory.

This paper presents a method to extend the topological invariant from closed to open systems. The scheme is based on a linear response theory developed here for open systems. By calculating the Hall conductance as a response to the adiabatic change of the Hamiltonian in momentum space, the topological invariant is proportional to the quantized Hall conductivity for the system in steady states.

## Results

To present the underlying principle of our method, we first extend the Bloch's theorem to open system, then derive the Hall conductance for open systems.

### Bloch's theorem and steady state

Take isolated electrons in a potential as an example, the Bloch's theorem for a closed system states that the energy eigenstate for an electron in a periodic potential can be written as Bloch waves. To extend this theorem from closed to open systems, we formulate this statement as follows. Consider an electron in a periodic potential 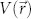 with periodicity 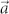, i.e., 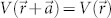. The one electron Schrödinger equation 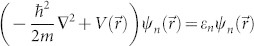
should also have a solution 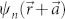 corresponding to the same energy *ε_n_*. Namely, 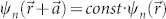. Here, *n* denotes the index for the energy levels, *m* is the mass of electron. Furthermore, the energy eigenstate can be written as, 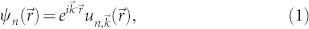
where 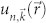 satisfies 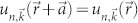 are the Bloch waves, 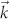 denotes the Bloch vector. Define a translation operator 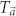 which, when operating on any smooth function 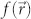, shifts the argument by 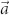, 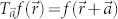. This operator can be explicitly written as 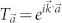. If 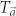 is applied to a Hamiltonian 
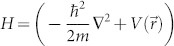
 with periodic potential 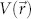, the Hamiltonian is left invariant, i.e., 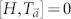.

Now we extend the Bloch's theorem from closed to open systems. Suppose that the density matrix *ρ* of the open system is governed by a master equation^12^, 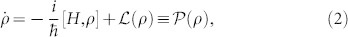
where 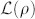 sometimes called dissipator describes the decoherence effect. In the absence of decoherence, we know that a key ingredient of the Bloch's theorem is 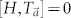. Thus, to preserve the translation invariant of the dynamics, it is natural to restrict the master equation to satisfy 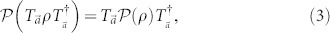
which is similar to 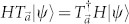 for a closed system. For a Lindblad master equation with decay rates *γ_j_* and Lindblad operators *F_j_*^12^, 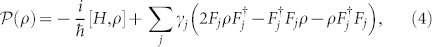
Eq. (3) leads to 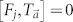 and 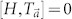 for any *j*. Consequently, when *ρ_ss_* is a steady state of the system, 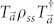 is also a steady state, since 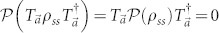.

The translation operator satisfying Eq. (3) preserve the decoherence-free subspace(DFS)^13^,^14^,^15^,^16^. DFS has been defined as a collection of states that undergo unitary evolution in the presence of decoherence. The theory of DFS provides us with an important strategy to the passive presentation of quantum information. The advantage of this translation-preserved-DFS is its possible applications into quantum information processing in the presence of decoherence.

Identifying the problem of energy eigenstates in closed system with the problem of steady states in open system, we formulate the Bloch's theorem of open system as follows. For an open system described by Eq. (2) with translation invariant map 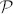, its steady state can be written as^7^,^8^, 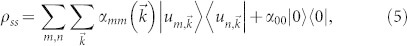
where 
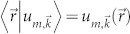
 are Bloch waves of the corresponding closed system, and |0〉 is the vacuum state. The coefficients 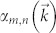 are independent of position 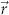, this fact can lift the limitation on the uniqueness required for steady states *ρ_ss_*. In other words, 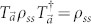 satisfy naturally in this situation. For the Lindblad master equation Eq. (4), 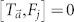 yields 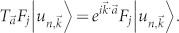


Thus, the Lindblad operators *F_j_* conserve the crystalline momentum 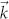 of the Bloch wave. This does not imply that the steady state has a well-defined crystalline momentum, since the steady state is a convex mixture of well-defined momenta states.

It is worth noticing that the Bloch's theorem of open system Eq. (5) relies on a postulate that the number of particles in the system is limited to below 1. When the number of particles is conserved, and consider the system having only one particle, the last term in Eq. (5) can be omitted.

In the following, we shall restricted our attention to open systems that possess translation invariance and preserve the TI phase. For this purpose, we need to specify how the dissipator is realized in physics. In an optical lattice setup, such a dissipative dynamics can be engineered by manipulating couplings of the lattice to different atomic species, which play the role of the dissipative bath^17^,^18^,^19^,^20^,^21^,^22^.

### Linear response formula for the Hall conductance

To derive the Hall conductance of an open system, we first develop a perturbation theory to calculate the steady state of the master equation Eq.(2). Perturbation theory is a widely accepted tool in the investigation of closed quantum systems. In the context of open quantum systems, however, the perturbation theory based on the Markovian quantum master equation is barely developed. The recent investigation of open systems mostly relies on exact diagonalization of the Liouville superoperator or quantum trajectories, this approach is limited by current computational capabilities and is a drawback for analytically understanding open systems.

In a recent work^23^, we have developed a perturbation theory for open systems based on the Lindblad master equation. In this approach, the decay rate was treated as a perturbation. Successive terms of those expansions yield characteristic loss rates for dissipation processes. In Ref. 24, instead of computing the full density matrix, the authors develop a perturbation theory to calculate directly the correlation functions. Based on the right and left eigenstates of the superoperator 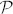, a perturbation theory is proposed^25^, the non-positivity issue of the steady-state may appear in this method due to truncations. Here, we apply the perturbation theory in Ref. 23 to derive the steady state. Instead of treating the decoherence as perturbation, a perturbed term in the Hamiltonian is introduced.

To present the main results of our method, we first consider a situation without decoherence, namely, for an open system described by the master equation, 
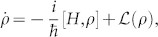
we have 
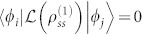
, where 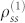 is the first order expansion of steady state, 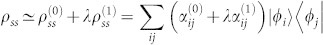, *λ* is the perturbation parameter from *H* = *H*_0_ + *λH*′, |*φ_i_*〉 is an eigenstate of *H*_0_ with eigenvalue *ε_i_*, *i* is the index for the eigenlevels. The steady state in this situation would be a diagonal matrix in the basis of energy eigenstates due to thermalization, i.e., 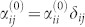 with *δ_ij_*, the Kronecker delta function. The expansion coefficients then reduce to, 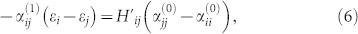
obviously, 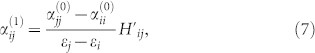
where 
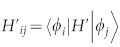
. To shorten the notation, here and hereafter, the perturbation parameter *λ* is included in 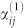. Namely, 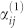 here and in the following equals the multiple of 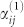 and *λ* in Eq. (38). Consider a x-direction weak electric field, 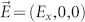, simple algebra yields(see Methods), 
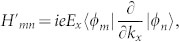
where *k_x_* is the x-component of 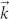, *e* is the charge of electron. Suppose the temperature is zero and the single filled band is the s-th Bloch band, i.e., all 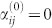 except 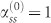, 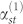 takes (*t* runs over the band indices), 
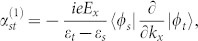
while 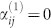 for other *i* and *j*. Collecting all these results, we have (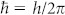, Planck constant) 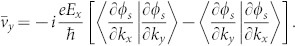


Here the fact that the contribution from the filled band is zero has been used. This is exactly the results in^10^,^11^,^26^ for closed systems.

**Figure 1:**
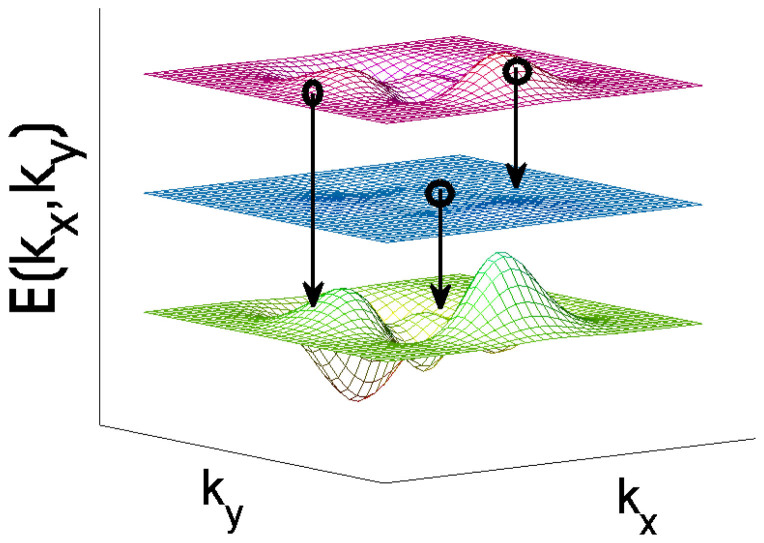
Illustration of the decoherence mechanism–decays from upper bands to the lowers.

Next let us consider what happens when there is a single steady band in the presence of decoherence. We refer the single steady band to that, with a fixed 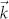, there is only a single energy eigenstate in the DFS. We denote this state by |*φ_s_*〉. In this case, the operator *F_j_* in Eq. (4) may takes, *F_j_* = |*φ_s_*〉〈*φ_j_*|. This describes a situation where all bands decay to the s-th band at rates of *γ_j_* with preserved momenta 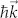, see Fig. 1. Straightforward calculation yields, 
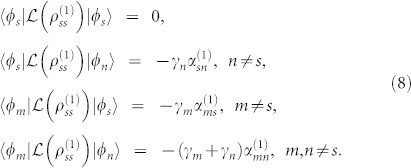
Substituting these equations into Eq. (35) and using 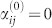 for any *i* and *j* except 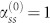, we arrive at 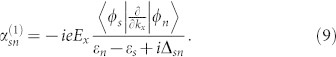
Here Δ*_sn_* is defined as Δ*_sn_* = *γ_n_* · (1 − *δ_ns_*), and 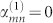 for *m* ≠ *s* and *n* ≠ *s*. For large energy band gaps, 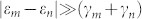, the coefficients approximately take, 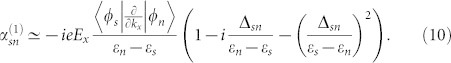
It is not trivial to extend the case of single steady band to two steady bands, as we shall show below. Denote the two steady bands by |*φ*_*s*_1__〉 and |*φ*_*s*_2__〉, respectively, a possible realization of the two steady bands is via a dissipator, 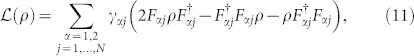
where we choose *F_αj_* = |*φ_sα_*〉〈*φ_j_*|, and *γ_αj_* denotes the decay rate. Following the same procedure as in the case of single steady band, we find 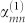 can be written in a form similar to Eq. (10), 
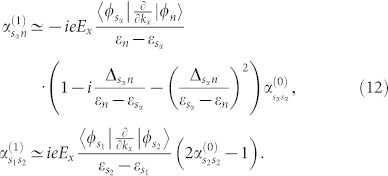
with 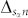 defined by, 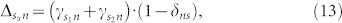
where *δ_ms_* = 1 when *s* = *s*_1_ or *s*_2_, otherwise it takes 0. Substituting 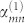 into the Hall current and supposing the current is zero in the absence of the external field, we find that the Hall current can be separated into two parts. The first part is independent of the decay rates and it can be written in terms of Chern number, while the second part takes a different form related closely to the dissipator. These two parts also manifest in the Hall conductivity discussed below, suggesting us to define a *topological* value called Chern rate for the system.

The Hall conductivity, defined as the ratio of the Hall current density *j_H_* and the electronic field *E_x_*, is therefore given by 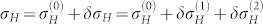. Here 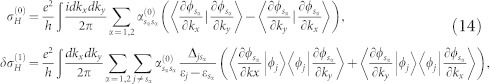

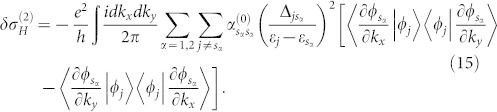


To derive these results, 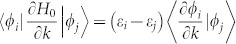 has been used. This is one of the main result of this work. It is worth pointing out that this result sharply depends on the decoherence mechanism. In fact, as we will show later in the two-band model, the Hall conductivity is not a mixture of Hall conductivities for various steady bands.

Assume 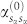 independent of *k_x_* and *k_y_*, the integral on the right hand side of 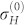, i.e., 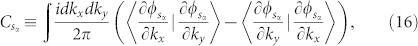
is nothing but the Chern number which takes integer values as pointed out in^26^. Then 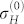 can be written as 
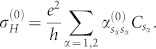

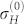
 is a weighted Chern number for the two steady bands. This term may not be an integer for a general open system, despite its topological origin. For 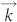-dependent 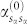, 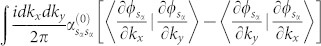 has been defined as the so-called Chern value^7^,^8^, which witnesses a topological non-trivial order present in the Berry curvature. It recovers the standard Chern number if the steady state is a pure Bloch state.

*δσ_H_* consists of two parts, 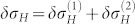. Here, 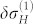 and 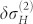 describe respectively the first order and second order corrections of the decoherence to the Hall conductivity. They can not be written in terms of Chern number in general, since both Δ*_mn_* and the energy gap depend on band index. Therefore, there is no topological invariance for the open system from the viewpoint of Hall conductivity, this is true even when the dissipation rates *γ_j_* and the band gaps are independent of band index, 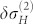 can be expressed in terms of Chern numbers in this case, but 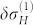 still can not. The Hall current given by *δσ_H_* characterizes the environmental activation of excited electrons in the bulk, and it is not zero in the regions outside the topological regime, where 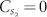. This can be found in Eq. (15).

These observations motivate us to define a topological value, to which we will refer as Chern rate, 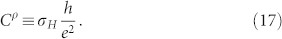
We adopt terminology Chern rate for the following reasons. Firstly, it possesses topological origin; Secondly, it may not take an integer for a general open system; Thirdly, it should differ from the Chern value defined in Ref. 7, 8, and in addition the Hall conductance is simply a multiple of the Chern rate and 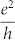. Of cause, the Chern rate returns back to the Chern number when the system is an isolated topological insulator. It is well known that Bloch's waves 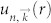 under time-reversal transformation take 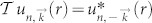, then the Berry curvature defined by 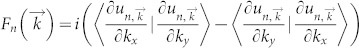 under the time-reversal transformation satisfies, 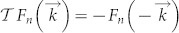. So, for system with time reversal symmetry, 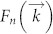 is an odd function of 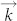. As a consequence, the Chern number for a time-reversal invariant system is zero, because the integral of an odd function over the whole Brillouin zone must be zero. This is not the case for second line in Eq. (15) that is an even function of *k*. This fact reflects that the second line in Eq. (15) may not be zero for a time-reversally invariant system, and hence the Chern rate loses partially its topological origin in this case. We will illustrate below that this non-topological term can be eliminated by properly designing *ε* and Δ in Eq. (18).

We now apply this formalism to derive a formula for Hall conductance in a two-band system. A decoherence mechanism different from this section is considered, namely the decoherence operator *F_j_* in the dissipator is not purely a Jordan block. This difference would manifest in the Hall conductivity, for example, the Hall conductivity is not a mixture of Hall conductivities for various bands.

### Applications of the formalism to a two-band model

We can apply the representation to develop a general formula for Hall conductance for a two-band system. Let us start with an effective Hamiltonian, 
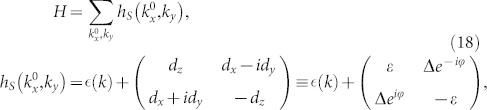
where 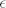 is the energy without couplings, it may take 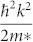 for the band electron with effective mass m*, and (*E*_0_ − *Dk*^2^) with constant *E*_0_ and *D* for the surface states of bulk Bi_2_Se_3_^27^. 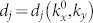 are the momentum-dependent coefficients which describe the spin-orbit couplings. *ε* = *d_z_*, 
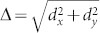
, 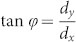, and 
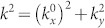
.

**Figure 2:**
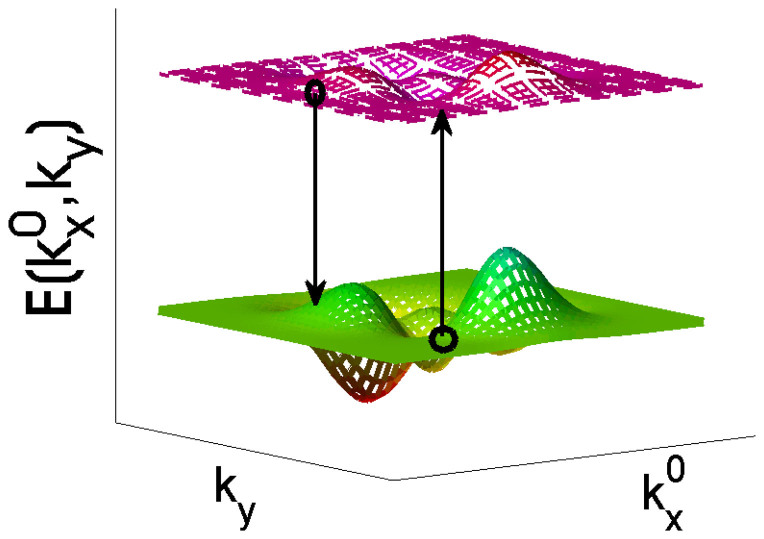
Illustration of the decoherence mechanism. It not only leads to a decay from the upper band to the low band but also a flip from the lower to the upper. Besides, it induces dephasings for each bands.

Consider phenomenally a dissipator, 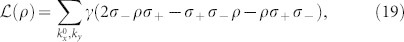
where 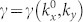 are momentum dependent decay rates, 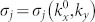, (*j* = +, −) are Pauli matrices. This dissipator describes a decay of the fermion from the spin-up state to the spin-down state with conserved momenta. It differs from those in the last section at that this dissipator does not describe decays from one band to the other, it instead characterizes the decay of the electron spin states, see Fig. 2.

Now we introduce a perturbation *λh*′ to Hamiltonian 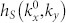, the total Hamiltonian with fixed 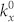 and *k_y_* is then 
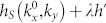
. Up to first order in *λ*, we write the steady state with fixed 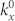 and *k_y_* as, *τ* = *τ*^(0)^ + *λτ*^(1)^. Tedious but straightforward calculations yield, 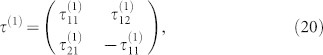
in the basis spanned by the eigenstates of 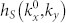, we have 
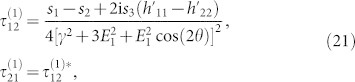
and 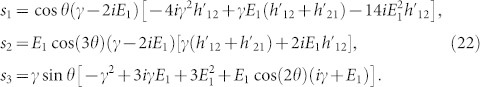
Here, 
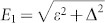
, 
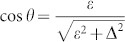
, 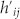, *i*, *j* = 1, 2 are matrix elements of *h*′ in the basis spanned by the eigenstates of 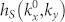. For more details, see Methods. The diagonal elements of *τ*^(1)^ is not listed here, since it has no contribution to the conductivity. In weak dissipation limit, *γ* → 0, we can expand 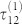 in powers of *γ*. To first order in *γ*, 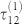 can be written as, 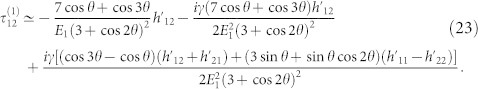
Assuming a weak electric field is applied along the x-direction and the corresponding vector potential is time-dependent, we find by simple algebra that, 
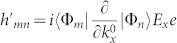
. Substituting these equations into the Hall conductivity and assuming *γ* independent of 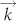, we have 
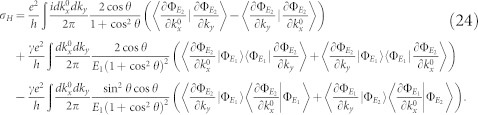
Discussions on the Hall conductivity are in order. The first integral describes a contribution of zeroth order in *γ*. It is different from the usual Hall conductivity of TIs with a single filled band |Φ_2_〉, the difference comes from the deviation of the steady state from the Gibbs states. Note that when Δ = 0, the first integral represents the usual Hall conductivity, the second and third integral represent a correction of dissipation to the Hall conductivity. We observe that the third integral vanishes with Δ = 0. In this case, the second integral reduces to, 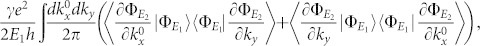
which is exactly the result in the last section for TIs with two bands. Noting that 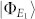 and 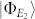 can be written in terms of *θ* and *φ*, 
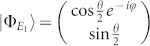
, 
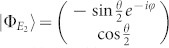
. We deduce the Hall conductance as, 
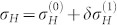
, 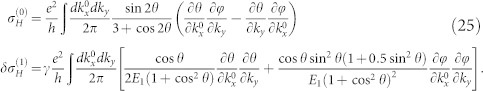
This equation is available for all two-band system described by the Hamiltonian in Eq. (18).

**Figure 3:**
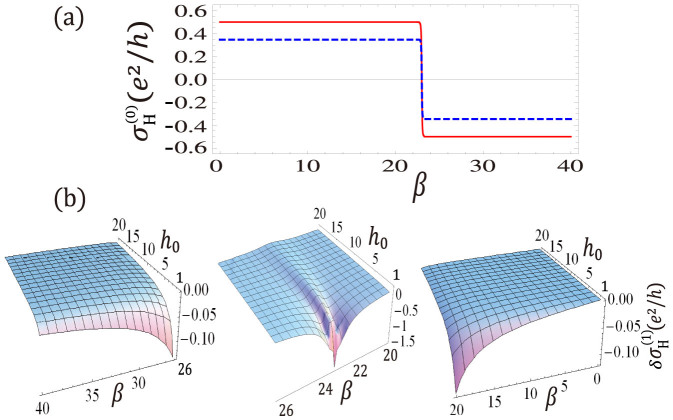
The zero-order and first-order conductivity 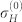 and 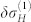 as a function of *β* (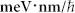) and *h*_0_ (in units of meV). Parameters chosen are, (a) *γ* → 0, 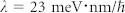, and (b)*γ* = 0.1 meV, 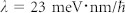. Note that 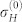 is independent of *h*_0_.

To be specific, we consider a two-dimensional ferromagnetic electron gas with both Rashba and Dresselhaus coupling, this system can be described by Hamiltonian Eq. (18) with *d_x_* = *λp_y_* − *βp_x_*, *d_y_* = −*λp_x_* − *βp_y_*, and *d_z_* = *h*_0_, here the momenta 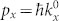 and 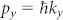. Using the formula Eq. (25) for the Hall conductivity, we calculate the Hall conductivity and show the numerical results in Fig. 3. Fig. 3(a) shows the zero-order Hall conductivity versus *β*. The red-solid line is for the closed system, while the blue-dashed line for the open system with *γ* → 0. It is interesting to notice that Hall conductivity of the open system with *γ* → 0 is different from that in closed system. This is easy to understand, the steady state of an open system is in general a mixed state, even though the decoherence rate is close to zero. Fig. 3(a) shows a phase transition at *β* = *β_c_* = *λ*, when *β* < *β_c_*, the Chern number of the closed system is 1, while for *β* > *β_c_*, the Chern number is −1. For open system, the phase transition can still be found from the Hall conductivity, even if the absolute value of 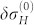 in the open system is smaller than that in the closed system. The first-order correction 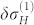 are negative on both sides of *β_c_*, as shown in Fig. 3(b), where we plot the first-order Hall conductivity as a function of *β* and *h*_0_.

The second concrete example is bulk Bi_2_Se_3_. The low-lying effective model for bulk Bi_2_Se_3_ can be formally diagonalized, which can be interpreted as the *K* and *K*′ valleys in the graphene^27^. For the valleys located at *K*, the effective Hamiltonian takes the same form as in Eq. (18) but with 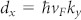, 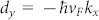, and 
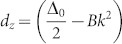
. A straightforward calculation shows that the term proportional to *γ* in the Hall conductivity is zero, this does not mean that the decoherence has no effect on the Hall conductivity. In fact, the decoherence leads the system to a mixed state, yielding the Hall conductivity, 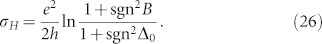
For *B* ≠ 0 and Δ_0_ ≠ 0, the Hall conductance is zero. For *B* = 0 and Δ_0_ ≠ 0, 
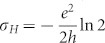
, and 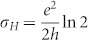 when *B* ≠ 0 and Δ_0_ = 0. This is different from the results of closed system^27^.

In the third concrete example, we apply the Hamiltonian Eq. (18) to model the two-dimensional lattice in a magnetic field^28^. The tight-binding Hamiltonian for such a lattice is written as, 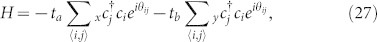
where *c_j_* is the usual fermion operator on the lattice, *t_a_* and *t_b_* denote the hopping amplitudes along the x- and y-direction, respectively. The first summation is taken over all the nearest-neighbor sites along the x-direction and the second sum along the y-direction. The phase *θ_ij_* = −*θ_ji_* represents the magnetic flux through the lattice. When *t_b_* = 0, the single band *E*(*k_x_*) is doubly degenerate. The term with *t_b_* in the Hamiltonian gives the coupling between the two branches of the dispersion. Consider two branches which are coupled by |*l*|–th order perturbation, the gaps open and the size of the gap due to this coupling is the order of 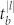. The effective Hamiltonian then take Eq. (18)^28^ with *φ* = *k_y_l*, 
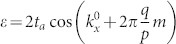
, and *δ* is proportional to (is the order of) 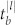. In terms of *d_x_*, *d_y_* and *d_z_*, the model takes, *d_x_* = *δ* cos(*k_y_l*), *d_y_* = *δ* sin(*k_y_l*), and 
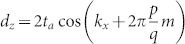
.

**Figure 4:**
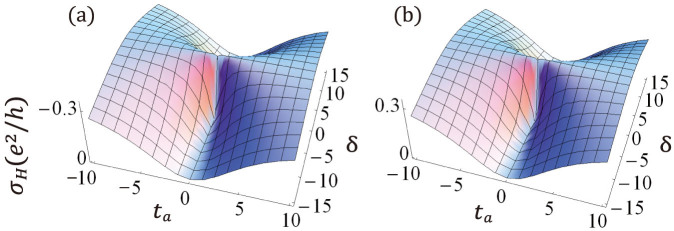
The conductivity *σ_H_* as a function of *δ* (in units of meV) and *t_a_* (in units of meV). Parameters chosen are *p* = 1, *q* = 4, *l* = 1, (a) *m* = 1, and (b) *m* = 2. Note that the sign of *σ_H_* in figures (a) and (b) are different. Further numerical simulations show that *σ_H_* depends only on the parity of *m*, i.e., figure (a) is for all odd *m*, while figure (b) for even *m*.

When applying the formula to this model, we can prove that 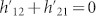 and 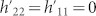. This can be done by examining the definition, 
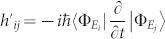
, and replacing 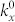 in Eq. (18) by 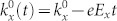. With this observation, the Hall conductance reduces to, 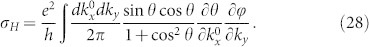
An interesting observation is that the correction of the decoherence to the Hall conductance is zero, this can be understood by examining Eq. (25), keeping in mind that *θ* depends only on 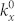 while *φ* only on *k_y_*. It is important to point out that the contribution from the steady state in the absence of external field was ignored in this section, this is reasonable that there has no current in the system when it reaches its steady state without external driving fields. In other words, we here only have interests in the current induced by the external fields, all of other contributions do not concern us. The dependence of the Hall conductivity on *δ* and *t_a_* is shown in Fig. 4. We find that *σ_H_* change sharply around *t_a_* = 0 except at *δ* = 0, but there is no phase transition at *t_a_* = 0 in the sense that the Hall conductance has a same sign for both positive and negative *t_a_*. The topological phase changes with the parity of *m*, when *m* is an odd integer, *σ_H_* < 0, whereas for even *m*, *σ_H_* > 0.

## Discussion

We have studied the Hall conductance of topological insulators in the presence of decoherence. After extending the Bloch's theorem from closed to open system, we have developed an approach to calculate perturbatively the steady state of the system driven by a perturbation. Then we apply this approach to derive the Hall conductance for the open system. We expand the Hall conductance in powers of dissipation rate, and find that the zeroth order covers the usual Hall conductance when the open system decays from a band to the others, whereas it can not return to the usual Hall conductance with a dissipator in the other form. The first order gives the correlation of the decoherence to the conductance, which vanishes for the two-dimensional lattice and contributes non-zero value to bulk Bi_2_Se_3_.

Generally speaking, the Hall conductance for open system can not be written as a multiple of a Chern number and a constant, or as a weighted sum of Chern numbers, in this sense, there is no topological invariant for open systems. The situation changes when a dissipator keeps the density matrix of the steady state in a diagonal form in a Hilbert space spanned by the instantaneous eigenstates of the Hamiltonian. Specifically, when the steady state takes, 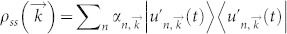 with 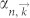 independent of time, and 
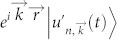
 denotes a wavefunction subject to the Hamiltonian, the Hall conductivity can be written as a weighted sum of Chern numbers. This is easy to find by expanding 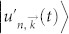 up to first order in the field strength and substituting the expansion into the Hall conductivity.

An interesting observation of this paper is that by properly designing the Hamiltonian, the decoherence effect on the Hall conductance can be eliminated in the two-band model. This observation makes the TIs immune to influences of environment and then support its application into quantum information processing.

The Kubo formula derived within the framework of linear response theory applies for equilibrium systems. Complementarily, we develop a formalism to explore the linear response of an open system to external field. Though we adopt a specific master equation to develop the idea, the general conclusion in this paper should be applicable to other open systems described by various master equations, in particular, for a system not in its equilibrium state.

## Methods

### Perturbation expansion of the steady state

We start with the master equation Eq.(2), and introduce a perturbed term *λH*′ to the Hamiltonian, 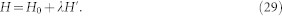
When applying the perturbation theory, we may separate the total Hamiltonian *H* in such a way that *H*_0_ is a proper Hamiltonian easy for obtaining the zeroth order steady state, while keep the perturbation part *λH*′ small. The steady state *ρ_ss_* can be given by solving 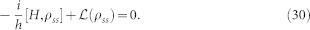
Up to first order in *λ*, the steady state can be expressed as, 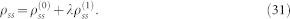
The zeroth order steady state 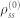 is then given by, 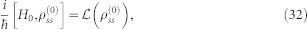
while the first order satisfies, 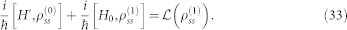
In a Hilbert space spanned by the eigenstates {|*φ_i_*〉} of Hamiltonian *H*_0_, *H*_0_|*φ_i_*〉 = *ε_i_*|*φ_i_*〉, the steady state can be written as, 
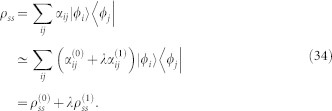
Substituting this expansion into Eq. (32) and Eq. (33), we obtain an equation for the coefficients 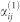, 
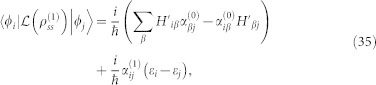
where 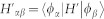, and 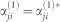. Assume the zeroth order steady state is easy to derive, the steady state up to first order in *λ* can be given by solving Eq. (35).

In order to derive the Hall conductance as a response to an external field, we consider the following idealized model: an non-interacting electron gas in an periodic potential 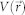. In the presence of a constant electric field 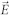 and when the field can be represented by a time-dependent vector potential, the system Hamiltonian takes^26^, 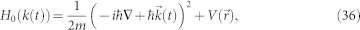
with 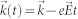. Taken the electric field in the x-direction, the y-component of the velocity operator in such a case is given by 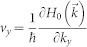
26. The y-component of the average velocity in the steady state is, 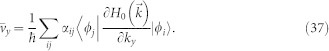
Up to first order in the perturbation *λ*, 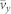 takes 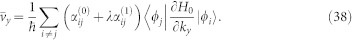
The Hall current density is given by, 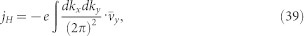
the Hall conductivity *σ_H_* is defined as the ratio of this current density and the electric field *E_x_*.

To calculate perturbatively the Hall current, we work in the weak field limit, *E_x_* ~ 0, this allows to use the adiabatic approximation to specify the perturbation Hamiltonian *H*′ induced by the adiabatic change of Hamiltonian 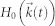 and calculate the perturbed steady state. We expand the density matrix in the basis of the energy eigenstates 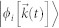 (the eigenstates of 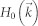) as, 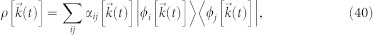
substituting this expansion into 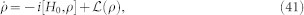
we have, 
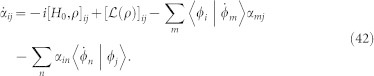
where for the sake of simplicity we shorten the notations as 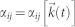 and 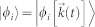. Notice that 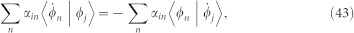
we obtain the Hamiltonian with a perturbation term *H*′, 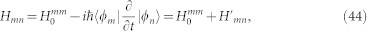
where, 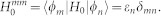


The Hamiltonian in Eq. (44) is the total Hamiltonian, which includes a part of zeroth order in *E_x_* and a term of first order in *E_x_*. In the following, we shall take *E_x_* small such that Hamiltonian *H*′ proportional to *E_x_* can be treated perturbatively.

### The zero-order steady state for two-band model

Solving the Schrödinger equation, *h_S_*|Φ*_E_*〉 = *E*|Φ*_E_*〉 with Hamiltonian Eq. (18), we can obtain the eigenenergies, 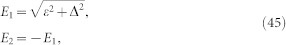
and the corresponding eigenstates, 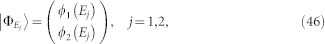
where, 
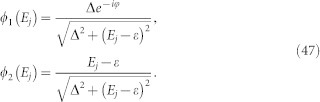
For the sake of simplicity, we transform the formalism into a Hilbert space spanned by the eigenstates of *h_S_*. Introducing 
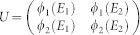
, we find that *h_S_* = *U H_dia_U*^†^ with 
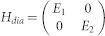
. Define *F* = *U*^†^*σ*_−_*U* and 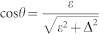, the elements of matrix 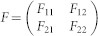 can be expressed as, 
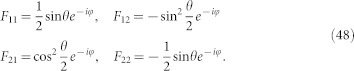
Collecting all these results, the master equation can be re-written as, 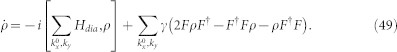
The steady state 
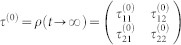
 with fixed 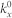 and *k_y_* can be given by solving, 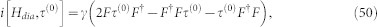
this gives rise to, 
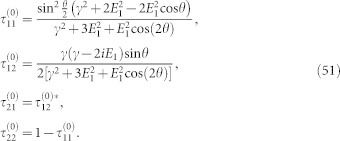
Here *τ*^(0)^ denotes the steady state without perturbations. In weak dissipation limit *γ* → 0, we find 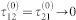, and 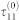 approaches 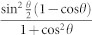. Obviously, in this limit, 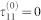 when Δ = 0, leading to the thermal state (ground state) at zero temperature. This observation suggests that the steady state under study is in general different from the Gibbs states, as a consequence, the Hall conductance would be different from that given by the Kubo formula.

## Author Contributions

X.X.Y. proposed the idea and led the study, H.Z.S., W.W. and X.X.Y. performed the analytical and numerical calculations, X.X.Y. and H.Z.S. prepared the manuscript, all authors reviewed the manuscript.
